# Prevalence and associated factors of oral frailty in elderly patients undergoing elective surgery: a secondary analysis of a cross-sectional study

**DOI:** 10.3389/fmed.2026.1748847

**Published:** 2026-04-21

**Authors:** Shizhao Wang, Keying Li, Yingchuan Zhang, Jiameng Yang, Rongtian Kang, Lining Huang

**Affiliations:** 1 Department of Anesthesiology, The Second Hospital of Hebei Medical University, Shijiazhuang, China; 2 Hebei Key Laboratory of Neurodegenerative Disease Mechanism, Shijiazhuang, China; 3 Key Laboratory of Clinical Neurology, Ministry of Education, Hebei Medical University, Shijiazhuang, Hebei, China

**Keywords:** associated factors, cross-sectional study, elective surgery, older adults, oral frailty, prevalence

## Abstract

**Background:**

Oral health has become a critical area of public health concern. Oral frailty refers to a significant decline in oral function and health, often manifested as impaired chewing and swallowing abilities as well as deterioration of oral structures. Oral frailty not only potentially affects postoperative recovery and quality of life but is also closely associated with various systemic health conditions. However, research on oral frailty in elderly patients undergoing elective surgery remains limited.

**Objective:**

This secondary analysis of a cross-sectional dataset aimed to investigate the prevalence of oral frailty and identify its associated factors among elderly patients undergoing elective surgery.

**Methods:**

This study is a secondary analysis of a cross-sectional study conducted in a tertiary general hospital in Shijiazhuang, Hebei Province, China, involving 300 patients undergoing elective surgery from October 2024 to May 2025. Data were collected using a general information questionnaire, the Oral Frailty Index-8 (OFI-8), number of natural teeth, the Mini Nutritional Assessment Short Form (MNA-SF), the FRAIL Scale, and the Rapid Cognitive Screen (RCS) to assess cognitive status. Chi-square tests and logistic regression analyses were performed to identify factors associated with oral frailty among elderly patients undergoing elective surgery.

**Results:**

The prevalence of oral frailty among elderly patients undergoing elective surgery was 43.0%. Age, female gender, frailty, reduced number of natural teeth, and preoperative cognitive dysfunction were identified as factors associated with oral frailty (all *P* < 0.05).

**Conclusion:**

The prevalence of oral frailty is high among elderly patients undergoing elective surgery. Healthcare providers should implement preventive management measures based on identified risk factors to control the occurrence and progression of oral frailty and to promote rapid postoperative recovery.

## 1 Introduction

As the global population continues to age rapidly (1), the number of elderly patients undergoing elective surgery has risen substantially. It is estimated that over 70 million older individuals undergo surgical procedures worldwide each year (2). In China, the proportion of older adults is also increasing significantly (3). Aging is accompanied by a progressive decline in physiological function and the accumulation of comorbidities, which increase susceptibility to perioperative complications (4). These age-related changes pose significant challenges for perioperative management.

In geriatric health, oral frailty has emerged as an important clinical concern. It is increasingly conceptualized as a multidimensional decline in oral function, encompassing impairments in dentition, chewing ability, articulatory oral motor skills, tongue pressure, and self-reported difficulties in eating and swallowing, as first described in epidemiological studies (5). Operationally, oral frailty is defined as the presence of deficits in three or more of these domains. This functional decline is often reflected in reduced tooth number, poor oral hygiene, and decreased oral performance (6). Growing evidence suggests that oral frailty is associated with adverse health outcomes, including cognitive impairment (7), osteoporosis (8), malnutrition (9), and increased fall risk (10), all of which may contribute to reduced quality of life and increased mortality.

Oral frailty is also closely associated with perioperative outcomes. In elderly surgical patients, it can increase the risk of nutritional complications, delay postoperative recovery, and elevate the risk of aspiration pneumonia, surgical site infection, and postoperative delirium, representing a significant threat to overall surgical outcomes (11–13). Despite its clinical relevance, perioperative oral frailty is often overlooked by healthcare providers.

Most previous studies have focused on community-dwelling older adults or patients with chronic diseases (14–16). In contrast, elderly patients undergoing elective surgery–a high-risk population facing considerable physiological stress–have received relatively little attention. Therefore, this study aimed to investigate the prevalence of oral frailty among elderly patients scheduled for elective surgery and to identify its associated factors. The findings may inform strategies for perioperative oral health management and guide the development of targeted interventions for this vulnerable population.

## 2 Materials and methods

### 2.1 Study participants

This study is a secondary analysis of data collected from a cross-sectional survey conducted at a tertiary hospital in Shijiazhuang, Hebei Province, China, between October 2024 and May 2025. The primary objective of the original study was to examine the relationship between oral frailty and preoperative cognitive dysfunction in elderly surgical patients. The study protocol was approved by the Ethics Committee of the Second Hospital of Hebei Medical University (approval number: 2024-R668) and registered at (ChiCTR 2400091189).

(1) Inclusion Criteria

Eligible participants were required to meet the following criteria: (1) aged ≥65 years, scheduled for elective surgery; (2) American Society of Anesthesiologists (ASA) physical status class II or III. These categories were included because they represent the majority of elderly patients undergoing elective surgery; ASA classification was further adjusted for in the statistical analysis to account for differences between groups; (3) no language communication barriers, with the ability to complete the questionnaire independently or with assistance from the researcher; and (4) provision of voluntary written informed consent.

(2) Exclusion Criteria

Exclusion criteria were as follows: (1) absence of preoperative data on oral frailty, nutritional status, or cognitive function; (2) history of severe psychiatric disorders (e.g., schizophrenia, bipolar disorder, or major depressive disorder), neurological diseases affecting cognitive function (e.g., epilepsy, Parkinson’s disease, or stroke), neuromuscular diseases (e.g., myasthenia gravis), or substance use disorders (including alcohol or drug abuse) that could interfere with study assessments.

### 2.2 Measurements

#### 2.2.1 General information questionnaire

The general information questionnaire was developed by the researchers based on the literature and study objectives. It included demographic and clinical variables such as age, gender, marital status, average monthly family income, place of residence, education level, living arrangement, frequency of weekly exercise, comorbidities, smoking and drinking history, number of remaining natural teeth, ASA classification, and cognitive function status. Data were collected by trained investigators on the day before surgery.

#### 2.2.2 Oral Frailty Index-8 (OFI-8)

The Oral Frailty Index-8 (OFI-8) was developed by Tanaka et al. to assess oral health-related behaviors and oral frailty (17). The scale includes eight items: (1) Difficulty eating hard foods compared to 6 months ago; (2) Occasional choking on tea or soup; (3) Use of dentures; (4) Symptoms of dry mouth; (5) Reduced frequency of outings compared to 6 months ago; (6) Ability to chew hard foods; (7) Brushing teeth at least twice a day; and (8) Visiting a dentist at least once per year. Scores range from 0 to 11, with higher scores indicating poorer oral health. A total score of ≥4 is indicative of oral frailty. The Chinese version of the OFI-8 demonstrated a Cronbach’s α coefficient of 0.61 (18).

#### 2.2.3 Nutritional assessment scale

Malnutrition is a known independent risk factor for postoperative complications and 1-year mortality. The Mini Nutritional Assessment–Short Form (MNA-SF) was used to assess nutritional status (19, 20). The total MNA-SF score ranges from 0 to 14, with 12–14 indicating normal nutritional status, 8–11 indicating risk of malnutrition, and 0–7 indicating malnutrition. Participants with a score <12 were classified as at risk of malnutrition or malnourished.

#### 2.2.4 Frailty scale

Frailty, characterized by multi-organ reserve loss, is prevalent among elderly patients and associated with adverse postoperative outcomes. The FRAIL Scale was used to assess frailty (21, 22). This five-item tool includes items on fatigue, resistance (ability to climb stairs), ambulation (ability to walk one block), illness (more than five diagnoses), and weight loss (≥5% unintentional weight loss). A score of ≥3 indicates frailty, and a score of 1–2 indicates pre-frailty.

#### 2.2.5 Rapid Cognitive Screen (RCS)

Cognitive dysfunction was assessed using the Rapid Cognitive Screen (RCS) (23). Scores of 8–10 indicate normal cognition, 6–7 indicate mild cognitive impairment (MCI), and 0–5 indicate dementia. In this study, MCI and dementia were grouped together as cognitive dysfunction.

### 2.3 Data collection method

Questionnaires were administered face-to-face and via Wenjuanxing, an online survey platform. Investigators provided one-on-one assistance to help patients complete the forms. Immediately after completion, questionnaires were checked for missing or unclear responses, which were corrected on-site. For participants with low education levels or presbyopia, the investigators read questions aloud and recorded responses.

### 2.4 Statistical analysis

Data were analyzed using SPSS version 26.0 (IBM Corp., Armonk, NY, USA). Continuous variables were assessed for normality using appropriate methods. Normally distributed variables are presented as mean ± standard deviation (SD) and were compared using independent-samples *t*-tests. Non-normally distributed variables are expressed as median (P25–P75) and were compared using the Mann–Whitney U test. Categorical variables are summarized as frequencies and percentages and were compared using Pearson’s χ^2^ test. Univariable logistic regression analyses were performed to explore the crude associations between each candidate variable and oral frailty. To identify factors independently associated with oral frailty, a multivariable binary logistic regression model was constructed. Variables included in the multivariable model were selected *a priori* based on clinical relevance, previous literature, and the study objective, rather than solely on statistical significance in univariable analyses. These variables included age, sex, physical frailty, number of natural teeth (<20 vs. ≥20), cognitive function, and other clinically relevant covariates assessed in this study. Adjusted odds ratios (ORs) with 95% confidence intervals (CIs) were calculated. Given the exploratory nature of this study, emphasis was placed on the magnitude and precision of the estimates rather than solely on statistical significance. A two-sided *P*-value < 0.05 was considered statistically significant.

## 3 Results

### 3.1 Participant characteristics

A total of 310 questionnaires were distributed, of which 302 were returned. After excluding incomplete or invalid responses, 300 questionnaires were included in the final analysis, yielding a valid response rate of 96.8%. Among the participants, 149 (49.67%) were male and 151 (50.33%) were female. The age ranged from 65 to 88 years, with a median age of 71.00 years (68–74). The distribution of surgical specialties was as follows: 183 cases (61.00%) in general surgery, 54 (18.00%) in urology, 31 (10.33%) in cardiothoracic surgery, 24 (8.00%) in gynecology, and 8 (2.67%) in orthopedics. Oral frailty was present in 129 participants (43.00%) and absent in 171 participants (57.00%). The baseline characteristics of the study population are summarized in Table 1.

**TABLE 1:** Basic characteristics of the elderly patients undergoing elective surgery (*n* = 300).

Variable	Category	Participants	Percentage (%)
Age	<70	149	49.67
	≥70	151	50.33
Gender	Female	151	50.33
	Male	149	49.67
Marital status	Single/divorced/ widowed	46	15.33
	Married	254	84.67
Residential area	Countryside	189	63.00
	City and town	111	37.00
Living arrangement	Living alone	34	11.33
	Not living alone	266	88.67
Malnutrition	No	271	90.33
	Yes	27	9.67
Frailty	No	251	83.67
	Yes	49	16.33
Surgical types	General surgery	183	61.00
	Urological surgery	54	18.00
	Thoracic surgery	31	10.33
	Gynecological surgery	24	8.00
	Orthopedic surgery	8	2.67
Monthly incomes	≤2000	175	58.33
	2000∼	72	24.00
	4000∼	53	17.67
Education level	Illiteracy	35	11.67
	Primary school	106	35.33
	Middle school	133	44.33
	High school and above	26	8.67
Number of chronic diseases	None	112	37.33
	One	111	36.67
	≥2	77	25.67
Frequency of weekly exercise	No	21	7.00
	1∼2 d	12	4.00
	≥3 d	267	89.00
Diabetes	No	242	80.67
	Yes	58	19.33
Hypertension	No	148	49.33
	Yes	152	50.67
Heart disease	No	269	89.67
	Yes	31	10.33
History of cerebral infarction	No	275	91.67
	Yes	25	8.33
Smoking	No	228	76.00
	Yes	72	24.00
Alcoholism	No	221	73.67
	Yes	79	26.33
ASA	II	222	74.00
	III	78	26.00
Number of teeth	≥20	173	57.67
	<20	127	42.33
Preoperative cognitive dysfunction	No	138	46.00
	Yes	162	54.00

### 3.2 Univariate analysis of oral frailty in elderly patients undergoing elective surgery

Univariable logistic regression analyses were conducted to examine the associations between individual variables and oral frailty. The results indicated that age, gender, marital status, nutritional status, frailty, number of natural teeth, frequency of weekly exercise, history of cerebral infarction, ASA classification, and cognitive dysfunction were significantly associated with oral frailty (*P* < 0.05). Detailed results of the univariate analysis are presented in Table 2.

**TABLE 2:** Univariate analysis of oral frailty in elderly patients undergoing elective surgery (*n* = 300).

Variable	Non-oral frailty (*n* = 171)	Oral frailty (*n* = 129)	*t/Z/χ^2^*	*P*
BMI	24.94 ± 3.08	24.82 ± 3.65	0.305^1)^	0.761
Age	104 (60.82)	45 (34.88)	19.784^2)^	<0.001
<70				
≥70	67 (39.18)	84 (65.12)		
Gender	74 (43.27)	77 (59.69)	7.926^3)^	0.005
Female				
Male	97 (56.73)	52 (40.31)		
Marital status	19 (11.11)	27 (20.93)	5.461^3)^	0.019
Single/divorced/widowed				
Married	152 (88.89)	102 (79.07)		
Residential area	104 (60.82)	85 (65.89)	0.812^3)^	0.368
Countryside				
City and town	67 (39.18)	44 (34.11)		
Living arrangement	15 (8.77)	19 (14.73)	2.596^3)^	0.107
Living alone				
Not living alone	156 (91.23)	110 (85.27)		
Malnutrition	164 (95.91)	107 (82.95)	14.145^3)^	<0.001
No				
Yes	7 (4.09)	22 (17.05)		
Frailty	161 (94.15)	90 (69.77)	31.994^3)^	<0.001
No				
Yes	10 (5.85)	39 (30.23)		
Surgical types	102 (59.65)	81 (62.79)	5.221^3)^	0.265
General surgery				
Urological surgery	31 (18.13)	23 (17.83)		
Thoracic surgery	21 (12.28)	10 (7.75)		
Gynecological surgery	15 (8.77)	9 (6.98)		
Orthopedic surgery	2 (1.17)	6 (4.65)		
Monthly incomes	94 (54.97)	81 (62.79)	1.867^3)^	0.393
≤2000				
2000∼	44 (25.73)	28 (21.71)		
4000∼	33 (19.30)	20 (15.50)		
Education level	14 (8.19)	21 (16.28)	5.874^3)^	0.118
Illiteracy				
Primary school	61 (35.67)	45 (34.88)		
Middle school	78 (45.61)	55 (42.64)		
High school and above	18 (10.53)	8 (6.20)		
Number of chronic diseases	67 (39.18)	45 (34.88)	1.741^3)^	0.419
Non				
One	65 (37.43)	46 (35.66)		
≥2	39 (22.81)	38(29.46)		
Frequency of weekly exercise	4 (2.34)	17 (13.18)	14.443^3)^	<0.001
No				
1∼2 d	9 (5.26)	3 (2.33)		
≥3 d	158 (92.40)	109 (84.50)		
Diabetes			0.000^3)^	0.986
No	138 (80.70)	104 (80.62)		
Yes	33 (19.30)	25 (19.38)		
Hypertension			1.171^3)^	0.279
No	89 (52.05)	59 (45.74)		
Yes	82 (47.95)	70 (54.26)		
Heart disease			1.046^3)^	0.306
No	156 (91.23)	113 (87.60)		
Yes	15 (8.77)	16 (12.40)		
History of cerebral infarction			4.907^3)^	0.027
No	162 (94.74)	113 (87.60)		
Yes	9 (5.26)	16 (12.40)		
Smoking			0.069^3)^	0.793
No	129 (75.44)	99 (76.74)		
Yes	42 (24.56)	30 (23.26)		
Alcoholism			1.105^3)^	0.293
No	122 (71.35)	99 (76.74)		
Yes	49 (28.65)	30 (23.26)		
ASA			10.974^3)^	<0.001
II	139 (81.29)	83 (64.34)		
III	32 (18.71)	46 (35.66)		
Number of teeth			35.913^3)^	<0.001
≥20	124 (72.51)	49 (37.98)		
<20	47 (27.49)	80 (62.02)		
Preoperative cognitive dysfunction			18.415^3)^	<0.001
No	97 (56.73)	41 (31.78)		
Yes	74 (43.27)	88 (68.22)		

^1)^, t; ^2)^, Z; ^3)^, χ^2^; BMI, body mass index; ASA, American Society of Anesthesiologists.

### 3.3 Multivariate analysis of oral frailty in elderly patients undergoing elective surgery

A multivariable logistic regression model was constructed to identify factors independently associated with oral frailty. Variables were selected based on clinical relevance and prior evidence rather than solely on univariable statistical significance. After adjustment, older age, female sex, physical frailty, having fewer than 20 natural teeth, and preoperative cognitive dysfunction were independently associated with higher odds of oral frailty. Specifically, older age was associated with increased odds of oral frailty (adjusted OR: 2.263, 95% CI: 1.229 ∼ 4.169). Female sex was also associated with higher odds compared with male sex (adjusted OR: 3.665, 95% CI: 1.641 ∼ 8.184). Patients with physical frailty had substantially higher odds of oral frailty (adjusted OR: 4.499, 95% CI: 1.665 ∼ 12.158). Having fewer than 20 natural teeth was strongly associated with oral frailty (adjusted OR: 4.974, 95% CI: 2.671 ∼ 9.260). In addition, cognitive dysfunction was associated with increased odds of oral frailty (adjusted OR: 1.896, 95% CI: 1.044 ∼ 3.443). The full results of the multivariable analysis are presented in Table 3.

**TABLE 3:** Logistic regression analysis of factors associated with oral frailty in elderly patients undergoing elective surgery.

Variable	OR	95% CI	*P*
Age	2.263	1.229 ∼ 4.169	0.009
Gender	3.665	1.641 ∼ 8.184	0.002
Frailty	4.499	1.665 ∼ 12.158	0.003
Number of teeth	4.974	2.671 ∼ 9.260	<0.001
Preoperative cognitive dysfunction	1.896	1.044 ∼ 3.443	0.036

OR, odds ratio; CI, confidence interval.

## 4 Discussion

Frailty is a multidimensional clinical syndrome characterized by a decline in physiological reserves across multiple organ systems, leading to increased vulnerability to stressors. With advancing age, this decline is often accelerated, compromising homeostatic mechanisms and increasing the risk of adverse health outcomes (24). Frailty has been widely recognized in geriatric medicine, and elderly patients undergoing elective surgery represent a particularly vulnerable population (25, 26). Despite this, perioperative assessments have traditionally focused on systemic conditions, while oral frailty–an important but often overlooked component of overall frailty–has received relatively limited attention. As a localized manifestation of systemic vulnerability, oral frailty may serve as an early indicator of functional decline. Previous studies have suggested that frailty, as assessed by tools such as the Hospital Frailty Risk Score, is associated with prolonged hospital stays and increased short- and long-term mortality in surgical populations (27). In this context, understanding oral frailty in elderly surgical patients may provide additional insights into perioperative risk stratification.

### 4.1 High prevalence of oral frailty in elderly patients undergoing elective surgery

In this study, the prevalence of oral frailty among elderly patients undergoing elective surgery was 43.0%. This is slightly lower than the 48.7% reported in a study from Anhui Province, China (28) and considerably lower than the 61.9% reported in hospitalized patients with chronic kidney disease (16). Conversely, it is higher than prevalence rates reported in community-dwelling elderly populations in China (30.2%) (29) and Japan (28.1%) (30), suggesting a gradient of oral frailty risk across different populations.

These differences may reflect variations in population characteristics and health status across study settings. Community-based cohorts typically include relatively healthier and more independent older adults, whereas hospitalized patients often have more advanced comorbidities and functional impairments. Elderly patients scheduled for elective surgery may represent an intermediate group, having undergone preoperative screening that excludes those with severe instability, yet still presenting with multiple age-related conditions. Additionally, regional differences in healthcare access, health literacy, and preventive care practices may contribute to variability in reported prevalence.

Taken together, these findings suggest that oral frailty is relatively common in the perioperative setting and may warrant consideration as part of comprehensive preoperative assessment. This observation is consistent with findings from recent systematic reviews and meta-analyses, which have reported a high overall prevalence of oral frailty among older adults and highlighted multiple contributing factors, underscoring the importance of early identification and targeted intervention (31, 32).

### 4.2 Factors associated with oral frailty

The factors associated with oral frailty identified in this study are broadly consistent with those reported in recent systematic reviews and meta-analyses (32). These findings further support the multifactorial nature of oral frailty, involving demographic, functional, and systemic health-related determinants.

#### 4.2.1 Age

Advancing age was confirmed as an independent risk factor for oral frailty in this study. Age-related degenerative changes in oral tissues–including reduced salivary secretion, enamel wear, periodontal atrophy, and thinning of the oral mucosa–can decrease chewing efficiency, impair swallowing coordination, and increase susceptibility to dental caries and periodontal disease, collectively contributing to the development and progression of oral frailty (33). Previous research indicates that oral frailty incidence is low in younger adults but accumulates over time, rising sharply after middle age (≥50 years) and peaking in older age groups (≥60 years), paralleling higher rates of periodontal disease (34). Our findings are consistent with this pattern and further suggest that advancing age may be an important marker of vulnerability in the perioperative context. However, as this study is cross-sectional, the temporal relationship between aging processes and oral frailty cannot be established.

#### 4.2.2 Gender

Female patients were found to have a higher risk of oral frailty compared with male patients, consistent with previous research (35). This increased risk may be related to postmenopausal estrogen decline, which predisposes women to osteoporosis and periodontal tissue atrophy, resulting in tooth mobility and loss (36). In addition, social and behavioral factors–such as differences in health-seeking behavior and symptom reporting–may also influence the observed association. These findings suggest that sex-related differences in oral health and function merit further investigation, particularly in perioperative populations.

#### 4.2.3 Physical frailty

Frailty prevalence among older adults has been reported to range from 4% to 59% (37, 38). Physical frailty is closely associated with oral frailty. Frail individuals often experience sarcopenia affecting the masticatory, tongue, and other oropharyngeal muscles, resulting in reduced chewing force, lower tongue pressure, and swallowing difficulties (39). Furthermore, frail patients frequently have limited exercise tolerance and decreased social engagement, which reduces functional stimulation of oral and orofacial muscles and accelerates oral functional decline (40). These findings highlight a potential link between physical and oral frailty.

#### 4.2.4 Number of natural teeth

Teeth, as essential organs for mastication, undergo age-related wear and functional decline. With advancing age, enamel regenerative capacity diminishes (41), and pulpal repair ability decreases (42). Having fewer than 20 natural teeth has been identified as a strong predictor of oral frailty (43). Tooth loss compromises chewing efficiency, reduces dietary diversity and adequacy, and increases the risk of preoperative malnutrition (44). It also alters occlusion, affects speech and facial appearance, and may decrease quality of life and social confidence. Moreover, a lower number of remaining teeth has been linked to postoperative delirium (45). These findings suggest that maintaining an adequate number of functional teeth may be relevant for overall health.

#### 4.2.5 Cognitive dysfunction

Cognitive dysfunction was also associated with higher odds of oral frailty in this study. Cognitive decline may impair oral health awareness and self-care ability. Individuals with cognitive impairment often demonstrate poor oral hygiene practices and may neglect early oral health problems, potentially accelerating oral functional deterioration (46). In addition, reliance on caregivers for oral care may introduce variability in care quality. These findings suggest that cognitive status may be an important factor to consider in oral health assessment among older adults. Further longitudinal studies are needed to explore the underlying mechanisms of this association.

## 5 Limitations

This study has several limitations. First, as the data were derived from a cross-sectional survey, causal relationships between potential risk factors and oral frailty cannot be established. Second, the sample was limited to elderly patients undergoing elective surgery at a single tertiary hospital in Shijiazhuang, Hebei Province, over a specific period, which may restrict the generalizability of the findings. Third, data were primarily self-reported, without objective validation, potentially introducing recall and social desirability biases, which could affect accuracy and reliability. Future studies should focus on developing and evaluating targeted interventions to reduce oral frailty in elderly patients. Large-scale, multicenter, longitudinal studies are needed to validate whether these interventions effectively reduce postoperative complications and improve long-term outcomes.

## 6 Conclusion

This study demonstrated a relatively high prevalence of oral frailty among elderly patients undergoing elective surgery. Advanced age, female gender, physical frailty, fewer than 20 natural teeth, and cognitive impairment were identified as independent risk factors. Oral frailty represents an important perioperative health concern that deserves attention comparable to systemic frailty.

Clinicians are encouraged to incorporate oral frailty assessments into routine preoperative screening. For high-risk patients, early, individualized, and multidisciplinary oral health interventions should be provided. Strengthening perioperative oral health management may improve nutritional status and functional capacity, thereby promoting better postoperative recovery and long-term outcomes.

## Data Availability

The original contributions presented in this study are included in the article/supplementary material, further inquiries can be directed to the corresponding author.
